# “From good hearted community members we get volunteers” – an exploratory study of palliative care volunteers across Africa

**DOI:** 10.1186/s12904-020-00545-w

**Published:** 2020-04-14

**Authors:** Carolin Clara Loth, Eve Namisango, Richard Antony Powell, Katharina Henny Pabst, Mhoira Leng, Mohamed Hamada, Lukas Radbruch

**Affiliations:** 1 Institute for Palliative Medicine, University of Bonn, Bonn, Germany; 2 African Palliative Care Association, Kampala, Uganda; 3 African Palliative Care Research Network, Kampala, Uganda; 4 MWAPO Health Development Group, Nairobi, Kenya; 5 Makerere University, Kampala, Uganda; 6 Cairdreas International Palliative Care Trust, Aberdeen, Scotland; 7 University of Edinburgh, Edinburgh, Scotland; 8 Al Azhar University Hospitals, Cairo, Egypt

**Keywords:** Volunteering, Africa, Palliative care, Lay-workers, Tasks, Motivation, Compensation, Recruitment, Training

## Abstract

**Background:**

Volunteers play a significant role in supporting hospice and palliative care in Africa, but little is known about the types of volunteers, their motivations and roles in service delivery.

**Methods:**

Palliative care experts from 30 African countries were invited to participate in an online survey, conducted in English and French, that consisted of 58 questions on: socio-demographics, the activities, motivation and coordination of volunteers, and an appraisal of recent developments in volunteering. The questionnaire was pre-tested in Uganda. Quantitative data was analysed descriptively with SPSS v22; answers on open-ended questions were analysed using content analysis.

**Results:**

Twenty-five respondents from 21 countries replied to the questionnaire. The typical volunteer was reported to be a female aged between 30 and 50 years. Volunteer roles included, among others: direct patient assistance, providing psychosocial / spiritual support, and assisting patients’ families. Respondents considered altruism, civic engagement and personal gain (for a professional career) as volunteers’ most significant motivational drivers. One in two respondents noted that recruiting volunteers is easy, and cooperation with the communities was often mentioned as helpful. Trainings mostly occurred before the first assignment, with topics covering the palliative care concept, care, psychosocial support and team work. Half of respondents described recent overall volunteering developments as positive, while the other half described problems primarily with financing and motivation. Most volunteers received transportation allowances or bicycles; some received monetary compensation.

**Conclusions:**

The findings show a wide range of volunteering in palliative care. We identified volunteers as typically 30–50 years old, non-professional females, motivated by altruism, a sense of civic engagement and personal gain. Palliative care services benefit from volunteers who take on high workloads and are close to the patients. The main challenges for volunteer programmes are funding and the long-term motivation of volunteers.

## Background

### Need for palliative care in Africa

There is a great need for palliative care in Africa as AIDS mortality remains high and rates of non-communicable diseases (NCD e.g., cancer or end-stage kidney disease) are rising [1, 2]. Although affordable antiretroviral treatment has been introduced to the region significantly since 2003 [3], there were still approximately 800,000 AIDS deaths in Africa in 2015 [4]. Cancer is frequently diagnosed late, and treatment options are often limited. Radiotherapy, for example, is estimated to cover only 34% of the need in Africa [5]. Another increasingly important NCD that imposes new challenges for palliative care provision is end-stage kidney disease [6]. Renal replacement therapy is rarely initiated due to the financial limitations of patients and institutions [6], and treatment is discontinued early for 90% of patients due to their inability to continue payments [7]. In these cases, the palliative care culture of open patient information on prognosis can support the difficult decision-making process.

### Health workforce crisis

The worldwide health workforce crisis, characterised by widespread global shortages, maldistribution of personnel within and between countries, migration of local health care professionals, and poor working conditions [8], is particularly pertinent in low-income countries [9, 10]. This acute crisis of human resources for health affects 57 countries, of which 36 are located in sub-Saharan Africa [11]. Moreover, although this region is inhabited by 11% of the world population and carries 24% of the global disease burden, it has only 3% of the world’s professional health care workers at its disposal [12].

### Palliative care provision in Africa

There is great heterogeneity in the provision of palliative care among African countries. The 2013 global atlas of palliative care showed a lack of organised activity in 28 African countries, while six (Kenya, Malawi, South Africa, Tanzania, Zambia and Zimbabwe) are ranked in the second highest category: “preliminary integration” into their health system [13]. Only one country, Uganda, is ranked in the report’s highest category (“advanced integration”).

Palliative care in Uganda was initiated in the early 1990s with the opening of Hospice Africa Uganda (HAU), offering care to patients with a day clinic, community outreach programmes and training courses. Today, palliative care is integrated into doctors’ and nurses’ training programmes at Mulago teaching hospital, and more hospices offer palliative care in a home setting. All these activities are largely supported by volunteers.

### Palliative care volunteers in Africa and worldwide

Volunteers’ roles and tasks differ considerably between low- and high-income countries. Case reports showcase the roles and value of volunteers, including the obstacles they face, in different settings, but there is a lack of comparative, regionally aggregated data.

Community volunteers in Uganda have been shown to work rather independently [14]. They act as a “bridge to the hospice” [15] by seeing patients regularly at their homes, and it is often the volunteers who identify new patients’ need for palliative care [14]. Their tasks include physical care, practical help and education [15].

A similar set of volunteer tasks exist in Kerala, India, including “taking anamnesis, performing simple nursing procedures, talking to patients and families, keeping the pharmacy, and visiting patients at home” [16]. In contrast, European volunteers’ tasks are mostly non-medical, e.g. by enabling patients to be outside [17]. Studies from other African countries have confirmed the value of volunteer work in hospice and palliative care. In Zimbabwe community volunteer training has been shown to be effective, but there is an identified need for close supervision and continuous training [18].

In terms of obstacles faced by volunteers, in South Africa financial insecurity and fear of infection have been identified as major considerations [19]. Financial insecurities have also been shown to be one of the main reasons why volunteers stopped engaging in a community volunteer programme in Rwanda [20]. Financial compensation is a common practice to help retain volunteers [20]. The most common challenges reported by Indian palliative care volunteers are lack of time to care for the high number of patients and emotional stress when working with paediatric patients [21].

In contrast, common problems Canadian volunteers encountered are “being underutilized, being placed with a patient too late in the patient’s illness, feeling undervalued by some members of the medical staff, and not being able to do more to help patients and their families” [22].

### Study rationale

Our understanding of volunteering in palliative care in Africa is currently derived from only these few countries. Published data suggests volunteering on the African continent differs in many aspects from volunteering in high-income settings, for which more data is available. However, as the African data covers only a small number of countries, it is unclear whether they are representative of palliative care volunteering for the whole continent.

This study aims to explore the scope of palliative care volunteering across Africa to increase our understanding of volunteers’ contributions in low-income settings. This will hopefully contribute to facilitating international dialogue and comparative analysis, policy making, the development of volunteering programmes and further research.

## Methods

Expert champions of palliative care (i.e. national association board members or pioneers organizing palliative care services) across Africa were invited to complete an online questionnaire on palliative care volunteering in the region.

### Identification of potential respondents

Candidates for the survey of national champions were identified by the authors in consultation with the African Palliative Care Association (APCA), a regional organisation cooperating with individuals and partner organisations in most African countries to strengthen the development of palliative care. The authors decided against strict eligibility criteria in order to include national champions of different professional backgrounds, especially in countries still in a capacity building developmental phase.

### Questionnaire development

The authors developed the study questionnaire in order to obtain information from both the African region—for the data presented in this article—and the European region. The European study’s results are analysed in a German-language medical thesis by one of the authors (KP) [17]; a separate international publication is planned.

During a six-week research visit of the lead author (CL) to Uganda, a draft questionnaire was pilot tested with either self-completion of its paper version questionnaires or with semi-structured interviews to obtain more detailed knowledge of interviewees’ opinions on the tool’s items. Local palliative care professionals (physicians, program directors, volunteer coordinators) and volunteers were interviewed. Additionally, palliative care professionals from francophone West African countries participating in a palliative care initiators course were interviewed.

After the pilot period, the initial draft was revised with a focus on clearer item wording and reduced completion time by omitting a number of questions. The final tool was translated into French by a palliative care professional fluent in both English and French, with both versions compared by a French palliative care professional, a process that complied with the translation policy of the European Association for Palliative Care (EAPC) [23].

The final questionnaire (Appendix 1) covered a broad range of topics using a mix of open and multiple choice (closed) questions. It specifically asked respondents to define volunteering from their perspective, and to provide information about hospice and palliative care services with volunteers in their country. Type and frequency of volunteer activities were assessed as well as volunteers’ roles and motivations. Volunteers’ motivations were assessed with a simplified version of the Inventory of Motivation for Hospice Palliative Care Volunteerism (IMHPV) [24] . The IMHPV’s five motivations are: altruism; civic engagement; self-promotion; leisure; personal gain.

Respondents were also asked about management aspects, such as volunteers’ compensation, their training and supervision and the initial recruitment process. Respondents’ views about the value of volunteering in palliative care, the development of volunteering in hospice and palliative care were included as well, as were their socio-demographic data.

In parallel with the national champions’ survey, similar questionnaires were used to collect data from volunteer coordinators and volunteers in the African countries. However, only five questionnaires were completed by coordinators, and eight by volunteers. Due to the small number, these questionnaires were not evaluated.

The survey phase started in January 2015 (English version) / March 2015 (French version) and ended in October 2015. Identified national champions were invited to take part in an online survey using SurveyMonkey. For those who could not complete the questionnaire online, there was the possibility of downloading the files and completing the questionnaire offline; these answers were then added to SurveyMonkey manually by the authors. Four reminders were sent out to invited respondents whose responses were pending. As an incentive, every respondent had the chance to win an iPad mini in a lottery draw after the data collection period.

### Data analysis

Datasets were exported from SurveyMonkey to SPSS v22, anonymised and merged for analysis. Quantitative data from the checkbox questions was not statistically analysed due to the small and non-representative sample size and heterogeneity. Absolute frequency numbers are therefore presented.

The original version of the IMPHV [24] was directed at volunteers themselves, whereas the questionnaire in this study was directed at national champions, asking about their perspective on volunteers’ motivations. The authors therefore modified the IMHPV, using a simple checkbox approach (yes/no = 1/0) instead of the Likert scale [1–5] to rate the statements. To be able to compare this study’s results with the original IMHPV findings, and thereby seek greater understanding of possible regional differences in the data, a standardised mean sample score was calculated for each motivation:

$$ \mathbf{Ms}= Standardized\ mean\ sample\ score=\frac{Mean\ sample\ score}{Maximal\ achievable\ sample\ score} $$

Qualitative data from open-ended questions was analysed thematically: content analysis was undertaken using pen and paper by one researcher (CL). Subsequently, the codes were discussed with a senior researcher (LR) until a consensus was achieved. The codes are presented in the qualitative data write-up in this article, with codes marked with italicised writing and followed by an example statement (French statements translated into English) and an identification tag (response number_country), where appropriate, in square brackets.

### Ethical considerations

The last data sheet, which was confidential and contained personal data, was analysed separately from the main data set to maintain anonymity. Information on the survey, data anonymisation and the evaluation methodology were included as background information for participants in the initial description of the online survey. We considered participation in the online survey to signify implicit consent for these data to be stored, evaluated and disclosed alongside other answers from the main data set, and therefore no separate informed consent form was included. It is unlikely that the identity each respondent will be revealed by the disclosed information, as there is generally more than one palliative care expert in a country. For statements with personal opinions, the country of origin did not add valuable information and was therefore not reported.

Ethical approval for the study was granted by the Uganda National Council of Science and Technology (registration number SS3503).

## Results

### Respondents’ country of origin and socio-demographics

Invitations were sent out to 54 national champions (38 English language questionnaires and 20 French language questionnaires; four national champions received questionnaires in both languages). Twenty-five national champions (response rate: 46.3%) from 21 countries (out of 30 countries approached; country coverage: 70%) responded, 16 questionnaires were completed in English and nine in French (see Table 1). Since respondents had the possibility of leaving questions unanswered, the total number of respondents differs per question.

**Table 1:** Respondents country of origin by language of questionnaire

English language respondents	Botswana, Egypt, Ghana, Kenya, Malawi [3], Mozambique, Namibia, Rwanda, Sudan, Swaziland, Tanzania, Uganda, Zambia, Zimbabwe
**French language respondents**	Benin, Burkina Faso, Burundi, Côte d’Ivoire, DRC [3], Rwanda, Senegal

Most (*n* = 18) national champions who responded had more than 10 years of professional experience. Approximately half (*n* = 10) of respondents were medical doctors, the others nurses (*n* = 6) or management staff (*n* = 4). The gender distribution was balanced (nine female, twelve male, four not reported).

### Definition of volunteering

Definitions of the term ‘volunteering’ predominantly included the absence of *financial compensation*, often stating that volunteers do not **expect** such a reward. A second recurring element was *altruism*, described either explicitly or by the absence of ulterior motives:

*“the service is provided out of a sense of love and passion”* [R13_BOTSWANA]*.*

*“deliver a service without pursuing any interest”* (translated from [R18_DRC])

Additionally, the concept of community – “*people who like to engage in the community*” [R6_RWANDA] – and the voluntary nature of activities performed by people “*out of their own free will”* [R2_GHANA], were repeatedly mentioned the definitions provided.

### Organisational structure

The majority of respondents (*n* = 15) considered the involvement of volunteers in hospice and palliative care a common practice in their country; however, all but one of these statements originated from Eastern Africa. Few francophone respondents considered this common practice in their country. Specific legal regulations for volunteering in palliative care were not cited by any national champion, and specific national standards for volunteering in palliative care were reported only once [R8_MALAWI].

Analysis of quantitative data on the organisational structure was not possible, since few respondents answered these questions and the numbers provided showed major discrepancies. Most national champions (*n* = 18) reported that coordination and supervision systems for volunteers were in place and run on both a peer-to-peer (*n* = 10) as well as a professional basis (*n* = 15).

### Socio-demographics of volunteers

In most African countries, the typical volunteer was described as a middle-aged (30–50 years) woman, who is not a health care professional. However, there were variations, such as in Egypt [R15_EGYPT], where only a few volunteers were reported as female, and half of volunteers as professionals. In some countries [R1_UGANDA, R24_BURUNDI], the majority of volunteers were described as younger than 30 years, while in Botswana most were reportedly over 50 years old [R13_BOTSWANA].

### Time spent by volunteers

Half (*n* = 9) of respondents reported that volunteers work roughly once per week, while a quarter (*n* = 6) reported more frequent activity and the other quarter less frequent activity (*n* = 5).

Most respondents stated volunteers can provide care from a patient’s point of diagnosis, while another frequent response was initiating volunteer care in patients’” *last months of life”*. For only two respondents it was noted that volunteers’ involvement always begins in the final phases (i.e., last days / weeks) of a patient’s life [R19_BENIN, R15_EGYPT].

### Type of volunteer activities

Respondents stated that volunteers were active in providing direct patient assistance, family assistance and psychosocial as well as spiritual assistance, with one respondent specifying medication adherence support: “*encourage to take ART [Anti-Retroviral Therapy], anti-TB [anti tuberculosis medication] and the like*” [R5_SWAZILAND]. Other areas of assistance included organisational support, fundraising and material support.

### Motivation of volunteers

Using the adapted IMHPV on volunteer motivation, the five motivational factors were ranked according to their relative importance: ‘altruism’ and ‘civil engagement’ were found to be the principal motivations, scoring a standardised mean sample score of M_s_ = 0.72 and 0.54, respectively (highest possible M_s_ = 1). They were followed by ‘personal gain’ (M_s_ = 0.38) and ‘leisure’ (M_s_ = 0.35). ‘Self-promotion’ was rated as least influential for motivation (M_s_ = 0.27).

### Volunteers’ roles

Volunteers were mostly regarded as part of the care team (*n* = 20), and none of the national champions regarded the volunteers as part of the family system. Their role was viewed as a professional one by some (*n* = 6), a lay person’s role by others (*n* = 7). Many national champions chose the ‘neither/nor’ option (*n* = 5).

### Volunteers’ compensation

Approximately half (*n* = 11) of national champions reported that volunteers received some kind of compensation for their efforts. The most common types of compensations reported were money (*n* = 8) and transportation (*n* = 8).

The amount of monetary compensation varied considerably, ranging from USD 2–160 per month; most respondents reported an equivalent of USD 20–60 per month per volunteer. Whilst payments seemed to be made by mostly non-governmental organisations (NGOs), in some countries the government provided the reimbursements [R6_RWANDA, R5_SWAZILAND].

Transportation compensation was primarily provided in the form of a bicycle donated to the volunteer (*n* = 8), followed by a transportation allowance as the second most popular method (*n* = 3). Individual motorised transport was reported twice, once by car and once by scooter.

Provision of meals (*n* = 3), goods (*n* = 3) (e.g., umbrellas and T-shirts [R8_MALAWI]) or medical care (*n* = 4) was also reported.

### Volunteer training

Most respondents (*n* = 12) reported the existence of training courses for hospice and palliative care volunteers. The most common reported form was a training course, completed before the first work assignment of the volunteer (*n* = 11), while others reported additional, regularly occurring training (*n* = 8). The duration of the typical training course was 1 to 2 weeks. An average training consisted of 47 h (median = 40 h) – ranging from 12 h in Sudan [R12_SUDAN], where all volunteers are clinical staff, to 112 h in Tanzania [R14_TANZANIA].

Frequent topics in the curricula of training programmes were ‘*Palliative care concept’,* physical care, psychological support and organisational processes. *‘Physical care’* included pain management and basic nursing care, along with education on nutrition. *‘Psychosocial support’* included family support, bereavement support, prevention, and spiritual and religious support. *‘Organisational processes’* included preparing volunteers to work with a professional team (e.g., team work, effective communication, data collection and referrals). Additional training for bereavement support was reported twice. For an illustrative of reported training topics see Table 2.

**Table 2:** Training topics – Detailed training curricula, as reported by two national champions, give an illustrative example of possible training concepts

Respondent	Training topics
**R11_MOZAMBIQUE**	introduction to the course; home visits/ social services; basic concepts on STI, HIV/AIDS; forms of HIV/AIDS prevention; nutrition education; chronic diseases more frequent; basic health care; education for the treatment; the burden of cancer/HIV; palliative care; registers and collecting data
**R3_KENYA**	Introduction to palliative care concepts; pain and symptom management; death and dying; breaking bad news; psychosocial support; bereavement; spiritual care; ethical issues in palliative care; the burden of cancer/HIV; home care; teamwork

### Benefits of volunteer involvement

The overall benefit of volunteers in hospice and palliative care entails the possibility of expanding the healthcare system’s reach both geographically and socially. The volunteers initiate and intensify contact between those in need and the health care system:

“*Volunteers make the identification of the patients in the community and forwards them to the hospital, but also ensure adherence to treatment making active search in coordination with the Health Unit*” [R11_MOZAMBIQUE].

Moreover, volunteers keep the health system operational by reducing the workload of professional staff, and by linking staff with patients, given they “*stay with the patients in the community, thereby understanding patients’ and family needs”* [R10_ZAMBIA].

Volunteers also contribute to the altruistic palliative care spirit:

*“palliative care is a labour of love”* (translated from [R22_DRC]).

### Challenges of volunteer involvement

The three main challenges faced with volunteering in hospice and palliative care were reported as: (i) lack of financial resources, (ii) lack of volunteer motivation and (iii) structural deficits. Lack of financial resources was reported as the absence of resources for volunteers themselves (e.g., training, stipends and coordination) and as a general struggle with resources required to implement palliative care. Challenges related to motivation were often linked to the lack of sufficient finances, with volunteer motivation undermined by the fact they cannot afford to work without any remuneration:

*“It’s a challenge to maintain them as they will need financial support for a living”* [R5_SWAZILAND].

Financial concerns reportedly could be aggravated by other demotivating factors:

“ *( … ) lack of communication about palliative care, existence of highly contagious diseases (Ebola)*” (translated from [R21_COTE D’IVOIRE]).

Structural deficits included the fact that in many African countries, palliative care services are offered by pioneers or organisations that are not well established:

“*The problem in Namibia, there is no well-established hospice*” [R4_NAMIBIA].

### Ease of volunteer recruiting

Ease of recruiting volunteers varied, with approximately half of respondents describing it as ‘*easy’* (*n* = 10) and the other half as ‘*not easy*’ (*n* = 9). A common recruitment strategy among the English-language respondents was to cooperate with local communities that could help identify candidates. As a Mozambique respondent noted:

“*it becomes easy because we work with community leaders and they are who makes the selection of volunteers within the community. The volunteers are part of the community where they work and have the confidence of the community*” [R11_MOZAMBIQUE].

One respondent noted that recruiting older volunteers was easier than recruiting younger ones:

“*Not so difficult [recruiting volunteers generally], but is difficult for youth to volunteer*” [R4_NAMIBIA].

### Limitations of volunteer activity

For most respondents there were explicit, sometimes legally required, limitations to hospice and palliative care volunteerism. This included medical procedures and nursing techniques:

“*not allowed to administer parenteral medication*” [R1_UGANDA].

“*the volunteers are not allowed to perform some nursing techniques that are the purview of nursing*” [R11_MOZAMBIQUE]*.*

Similarly, the distinction between lay volunteers and professional health care workers led to the same limitations for key activities *“unless the volunteer is a doctor or nurse*” [R3_KENYA].

For some respondents, there were no limitations to voluntary work other than the volunteer’s competence:

“*(...) they can work according to their competence”* (translated from [R19_BENIN]).

### Changes in volunteer activity

Asked about changes occurring in hospice and palliative care volunteering in recent years, half of the respondents (*n* = 6) reported negative changes, the other half positive ones (*n* = 7); two respondents stated there had not been any major changes. Negative changes mostly concerned recruitment and retention issues caused by (i) the unavailability of incentives, (ii) volunteers struggling with the cost of living and (iii) a decline in working morale caused by the perceived receding impact of the HIV/AIDS epidemic.

Positive changes included patients and communities benefiting from volunteers’ activities and knowledge:

*“Many patients had someone to talk to most times*” [R8_MALAWI]*.*

“*The community health workers also support preventive health initiatives in their respective communities*“ (translated from [R20_RWANDA]).

Another respondent noted it had become easier to recruit local volunteers:

“*In the past volunteering was from abroad … but now the biggest positive change is that ( … ) from good hearted community members we get volunteers*” [R14_TANZANIA].

It was also reported that volunteers make a decentralisation of hospice service provision possible. This allows more people from a wider geographic area to access hospice and palliative care:

“*Today it is easy to decentralise the concept of palliative care in the community because of the presence of volunteers and we reduced the flow of patients in the hospital*” [R6_RWANDA].

### Possibilities for external support

The national champions identified possibilities for external support on both the organisational and the coordinator level. On the organisational level support with advocacy, funding and promotion of research activity were suggested. For support of volunteer coordination, the development of (i) training curricula, (ii) coordination and (iii) supervision methods and motivational strategies was suggested. Another proposal was to organise awards for the most outstanding volunteers.

## Discussion

This discussion will focus on two topics we identified as key findings because of their relevance for palliative care providers: volunteers’ motivation and the future of volunteering in Africa. The limitations of the study will also be discussed.

### Volunteer motivation

The principal motivations of volunteers to participate in hospice and palliative care were, according to survey results, ‘altruism’ and ‘civic engagement’. This is comparable to Claxton-Oldfield et al’s [25] original IMPHV results among British and Canadian hospice and palliative care volunteers, who also found these motivations to be predominant. ‘Personal gain’ (for professional development) was more prominent in the African sample, while ‘leisure’ and ‘self-promotion’ were emphasised less. Claxton-Oldfield et al. contended that the limited influence of personal gain in their samples was probably due to demographic profiles: the samples were dominated by elderly (i.e ≥ 60 years) in a resource-rich setting, for whom material or professional gain was probably not an imperative. In contrast, our study found volunteers to be younger, mostly 30–50 years, belonging to a demographic group that is part of the work force and which may have to provide for a family. In the low-income setting of most African countries, it is not surprising that every activity, including volunteering to care for the severely ill and dying that is primarily triggered by altruism, is viewed also through the lens of a potential source of income. To run a successful volunteer programme and to retain skilled volunteers, it is crucial to take their motivations into account.

Strategies directed at volunteers for whom professional development is an important factor include

(i) internship-inspired volunteer programmes,
(ii) microfinance credits and
(iii) financial compensation.

Examples of all three strategies have been reported from African settings:

(i) During the pre-test research period in Kampala two university students who had volunteered full-time for a specified period of time were interviewed. One of them had already completed a full-time placement and continued his voluntary engagement parallel to his studies at medical school. The other had started to volunteer as part of an internship in the human resource department of the organisation. After his exams, he continued to work as a full-time volunteer while waiting for a job opportunity.
(ii) A nationwide community health system in Rwanda, in which community-based volunteers play an important role, offers access to microfinance credits to its volunteers. These credits support volunteers to start their own businesses [20] .
(iii) Establishing financial compensation for volunteers is a method widely discussed in the literature on health care volunteering in the African region. Our results indicate that financial compensation for volunteers exists in approximately a third (8 out of 23) of the countries represented. Amounts received by volunteers vary extremely, between USD 2–160 per month. Respondents’ comments showed that an important distinction has to be made between money covering only transportation costs (which will be referred to as *expenses*) and money exceeding the transport costs (which will be referred to as *stipends*).

Jack et al. [14] provided a good example for *expenses* in their study of a hospice and palliative care volunteer programme in Uganda: They reported that many of the first volunteer cohort left the programme due to the costs they incurred travelling to see patients.

Bertrand-Farmer [20], who also reported on the microfinance programme, makes a case for stipends. She explains that when relying on a volunteer to care for patients’ needs, the volunteer’s need to pay for food and children’s school fees has to be considered. A similar observation has been made in our survey, when one respondent stated that volunteers need financial support to make a living.

The national champions’ definitions of volunteering in our survey made it clear that the stipends given to volunteers are intended to be enabling in nature and not a salary; recurring wording was that volunteers offer a service “*without expecting*” monetary compensation.

Maes’ [26] consensus analysis of the motivation of AIDS care volunteers challenges this perception of volunteer stipends. While NGOs emphasise that volunteers are driven by altruism and intrinsic motivation, Maes contends that volunteers expect to receive food stipends regularly and are motivated by the chance of raised compensation packages in the future and the hope of job opportunities. He concludes that volunteers see the food stipend as a salary, rather than an enabling compensation.

### Future of palliative care volunteering in Africa

Respondents to this survey reported that volunteers are an essential vehicle of the palliative care spirit, and that volunteers offer the patients someone additionally to talk to. Moreover, there is a more tangible benefit of volunteer involvement: more patients can be cared for, as community volunteers help decentralise care provision and reduce the workload of professional staff. Given these benefits, volunteer involvement qualifies as a strategy for the development of palliative care in the region. However, this study showed that volunteering in palliative care is still not common in many African countries, especially in francophone (Western) Africa.

Establishing and running volunteer programmes requires funding. Indeed, the challenge most often mentioned by our respondents, and thus probably most relevant for the future of volunteer programmes in Africa, is funding. International NGOs are a common source of funding, but these funding streams are usually unpredictable and project-specific. An alternative approach that could be explored are community-based financial schemes. A best practice model is the Neighbourhood Networks for Palliative Care operating in Kerala, India [27], using micro-funding and community engagement to support volunteer networks. Another alternative is a public health approach with a government lead as in Rwanda, where over 90% of the population have health insurance that covers hospitals, health stations and volunteers working as community health workers [28].

Recruiting volunteers and keeping them motivated was another great challenge respondents experienced. Financial insecurity certainly is a major impeding factor for volunteering in general. A hindrance more specific to volunteering in palliative care is the fear of infection, cited by a respondent from Western Africa in connection with the Ebola virus. The risk of infection demonstrates the importance of appropriate training for volunteers. Volunteers who are trained in prevention of transmission of infections cannot only protect themselves, but also spread skills and knowledge within their communities. One respondent described this combined effect of volunteers who are not only engaging in palliative care, but also supporting preventive health interventions.

Digitalisation was not mentioned by any respondent as already leading to changes in volunteer involvement. Discussions at the 2016 APCA conference in Kampala showed, however, significant interest in new possibilities arising from technological innovations, especially for keeping contact with patients, families and volunteers in rural areas.

### Study limitations

National champions are an indirect source of information on volunteers. In countries with multiple respondents, contradicting answers indicated insufficient knowledge or lack of consensus. However, the national champions we approached as respondents for the survey were selected in close cooperation with APCA and other major stakeholders, and thus represent the best available sources of country-specific information. Due to the early stages of palliative care development in many countries, those national champions do not have purely administrative jobs, but work in regular contact with volunteers. Furthermore, most respondents identified themselves as professionals with more than 10 years of experience. Still, triangulation of such champions’ perspectives on volunteering with information obtained from volunteers themselves is an imperative for future research.

The regional distribution of the 21 African countries represented was skewed, with an over-representation of respondents originating from Eastern Africa (*n* = 11). This might have been caused by a bias towards the Eastern African countries because of a higher development of volunteer work in hospice and palliative care and also palliative care development in general. The questionnaire was provided in a French version to facilitate participation from francophone countries. The translation to French could have had an impact on the results’ validity, but to minimise this impact a pre-tested translation method was used. As the questionnaire was not provided in Arabic, linguistic barriers might have been the cause for lack of contributions from Northern African countries (*n* = 1).

The comparison of the African sample with data from Canadian and British volunteers in the IMHPV has to be interpreted with caution, as the presented sample used an indirect proxy perspective from national champions on volunteering, and did not obtain information from volunteers themselves. Validating the IMHPV through factor analysis of surveys in different African settings with a higher number of participants would be interesting future research. Financial reimbursement as a possible motivation of volunteers should then be added as a new category, to study the influence of commonly practiced compensations.

## Conclusions

This survey demonstrated significant diversity in volunteering in hospice and palliative care in African countries. In most countries, the typical volunteer was described as a female, 30–50 years old, who is not a health care professional. She was thought to be motivated by altruism, a sense of civic engagement and by potential gain for her professional career, rather than by the need for leisure activity or self-fulfilment. In some countries, a volunteer might receive financial compensation, either covering expenses or acting as a stipend to cover part of the cost of living. Volunteers’ activities commonly included direct patient assistance, providing psychosocial/spiritual support, and assisting patients’ families. It can be concluded that having “good-hearted people from the community” as volunteers allows for expansion of palliative care services and is a major asset in hospice and palliative care.

## Supplementary information

**Additional file 1.** Questionnaire – full questionnaire as available to English language participants of the survey.

## Data Availability

The datasets used and/or analysed during the current study available from the corresponding author on reasonable request.

## Abbreviations

APCA: African Palliative Care Association
EAPC: European Association of Palliative Care
IMHPV: Inventory of Motivation for Hospice Palliative Care Volunteerism
NGO: Non-governmental organisations
