# Supplementary material for: “From good hearted community members we get volunteers” – an exploratory study of palliative care volunteers across Africa
Source: BMC Palliat Care. 2020 Apr 14;19:48. doi: 10.1186/s12904-020-00545-w (PMC7158085; doi:10.1186/s12904-020-00545-w)
Supplement: Supplementary file 1 — Additional file 1. Questionnaire – full questionnaire as available to English language participants of the survey. [file 12904_2020_545_MOESM1_ESM.docx]

| **I** | **General information** | | | | | | | | | | | | | | |
| --- | --- | --- | --- | --- | --- | --- | --- | --- | --- | --- | --- | --- | --- | --- | --- |
|  | *Please refer to volunteers/ voluntary services* ***in your country*** *when answering the questions. This survey is concerned with* ***non-professional volunteering****. Health care professionals or other professionals offering their services as volunteers are targeted only where specifically mentioned.*  *Sometimes we ask for numbers or percentages, please feel free to estimate!*  *Where multiple answers are not possible and indicated, we kindly ask you to choose the answer that would be true in most cases.* | | | | | | | | | | | | | | |
| 1. | Which country do you work in? | | | | | | | | | | | | | | |
|  |  | | | | | | | | | | | | | | |
| 2. | How would you define the term ‘volunteering’? | | | | | | | | | | | | | | |
|  |  | | | | | | | | | | | | | | |
| 3. | Would you consider the involvement of volunteers in hospice and palliative care as common in your country? | | | | | | | | | | | | | | |
|  |  | Yes | | | | |  | No | | | | | | | |
| 4. | How are volunteer activities organised in hospice and palliative care?  *(Multiple answers possible)* | | | | | | | | | | | | | | |
|  |  | In separate services/ organisations for volunteers | | | | |  | There are no set organisational structures for volunteers in my country | | | | | | | |
|  |  | In hospice or palliative care services | | | | |  | Other *(please specify)*: | | | | | | | |
| 5. | Please estimate the number of organisations/ services for volunteers in your country: | | | | | | | | | | | | |  | |
| 6. | Please estimate the average number of volunteers participating in each service: | | | | | | | | | | | | |  | |
| 7. | In which hospice and palliative institutions can volunteers provide their support in your country? *(Multiple answers possible)* | | | | | | | | | | | | | | |
|  |  | | | | | | | | | | | | | | |
|  |  | Palliative care unit in a hospital | | | | |  | Inpatient hospice | | | | | | | |
|  |  | Palliative home care services (with predominantly professional paid staff) | | | | |  | Hospice home care services (with predominantly volunteers) | | | | | | | |
|  |  | Day hospice | | | | |  | Other *(please specify)*: | | | | | | | |
| 8. | Please estimate how many of the hospice and palliative care volunteers are female: | | | | | | | | | | | | | | |
|  |  | |  | Hardly any |  | A few | | |  | Many |  | Most |  | | Almost all |
| 9. | Please estimate how many of the hospice and palliative care volunteers are… | | | | | | | | | | | | | | |
|  |  | | | | | | | | | | | | | | |
|  | ...< 30 years old? | |  | Hardly any |  | A few | | |  | Many |  | Most |  | | Almost all |
|  | ...30-50 years old? | |  | Hardly any |  | A few | | |  | Many |  | Most |  | | Almost all |
|  | …> 50 years old? | |  | Hardly any |  | A few | | |  | Many |  | Most |  | | Almost all |
| 10. | Please estimate for how long hospice and palliative care volunteers provide voluntary services on average per patient in your country:  *(Multiple answers possible)* | | | | | | | | | | | | | | |
|  |  | | | | | | | | | | | | | | |
|  |  | Last days of life | | | | |  | Last months of life | | | | | | | |
|  |  | Last weeks of life | | | | |  | From diagnosis on | | | | | | | |
|  |  | Other (*please specify*): | | | | |  |  | | | | | | | |
| 11. | Please estimate how often hospice and palliative care volunteers provide their service:  *(Multiple answers possible)* | | | | | | | | | | | | | | |
|  |  | | | | | | | | | | | | | | |
|  |  | More than once a week | | | | |  | Roughly once a week | | | | | | | |
|  |  | Once or twice per month | | | | |  | Several times per year | | | | | | | |
|  |  | Less than several times per year | | | | |  | Just once | | | | | | | |
|  |  | Other *(please specify)*: | | | | | | | | | | | | | |
| 12. | Please estimate: How many hours does the typical hospice and palliative care volunteer work per month? | | | | | | | | | | | | |  | |
| 13. | Please estimate: How many different patients does the average hospice and palliative care volunteer care for each month? | | | | | | | | | | | | |  | |
| 14. | How many volunteers in hospice and palliative care exist in total in your country (if known)? | | | | | | | | | | | | |  | |
| 15. | Please estimate: Which percentage of patients in hospice or palliative  care have volunteers involved in their care? | | | | | | | | | | | | | % | |
| 16. | Please estimate the percentage of volunteers (from the total number of volunteers in your country) that are active in the following types of hospice or palliative care facilities: | | | | | | | | | | | | | | |
|  |  | | | | | | | | | | | | | | |
|  | Palliative care ward: | | | | | | | | | | | | | % | |
|  | Inpatient hospice: | | | | | | | | | | | | | % | |
|  | Day hospices: | | | | | | | | | | | | | % | |
|  | Palliative home care: | | | | | | | | | | | | | % | |
| 17. | If no volunteers work in any of these institutions, could you provide a reason for this? | | | | | | | | | | | | | | |
|  |  | | | | | | | | | | | | | | |

| **II** | **Tasks and roles** | | | | | | | |
| --- | --- | --- | --- | --- | --- | --- | --- | --- |
| 1. | Which of the following activities are performed by volunteers in your country? | | | | | | | |
|  |  | | Direct patient assistance | |  | | | Psychosocial/ spiritual assistance |
|  |  | | Assistance to the patient’s family | |  | | | Organizational support for the service: |
|  | Other *(please specify)*: | | | | | | | |
| 2. | What do you think drives people to become a hospice or palliative care volunteer?  *Please take your time reading the following answers and tick all the relevant fields.* | | | | | | | |
|  |  | | | | | | | |
|  |  | Help others cope with death and dying. | |  | | | People tend to look favorably on volunteers. | |
|  |  | Help ease the pain of those living with a life threatening illness. | |  | | | Feel better about oneself. | |
|  |  | Help those who are facing death. | |  | | | Enjoy having something to do with ones time. | |
|  |  | The wish to make others comfortable in life, as well as until death. | |  | | | Meet other people. | |
|  |  | Support the philosophy of palliative care. | |  | | | Activity to focus on others instead of oneself. | |
|  |  | Believe that everyone should give something back to the community. | |  | | | Hobby. | |
|  |  | Believe that volunteering is a required part of community service. | |  | | | Exciting/involving work. | |
|  |  | Responsibility to help others. | |  | | | Help with future goals | |
|  |  | Generally people are obligated to provide service to the towns they live in. | |  | | | Requirement to fulfill ones involvement in another activity. | |
|  |  | Improve the image portrayed to family, friends, and society. | |  | | | Get experience in a “helping profession”. | |
|  |  | Like the attention one gets when volunteering. | |  | | | Get a foot-in-the-door for potential employment. | |
|  |  | Like being needed. | |  | | | Work in the medical field. | |
|  |  | Other *(please specify)*: | |  | |  | | |
|  |  | Here is place for your comment on what drives volunteers | | | | | | |

| 3. | How do volunteers (in your opinion) understand their role related to patients and their family members? | | | | | | | | | |
| --- | --- | --- | --- | --- | --- | --- | --- | --- | --- | --- |
|  |  | | | | | | | | | |
|  | a) *(Please choose one option only)* | | | | | | | | | |
|  |  | |  | Confidential friend | |  | | Neither nor |  | Stranger |
|  | b) *(Please choose one option only)* | | | | | | | | | |
|  |  | |  | Expert | |  | | Neither nor |  | Layperson |
|  | c) *(Please choose one option only)* | | | | | | | | | |
|  |  | |  | Part of the care team | |  | | Neither nor |  | Part of the family system |
|  | d) *(Multiple answers possible)* | | | | | | | | | |
|  |  | |  | Coordinator of hospice or palliative care | | | | | | |
|  |  | |  | Gatekeeper for hospice or palliative care | | | | | | |
|  |  | |  | Advocate against doctors | | | | | | |
|  |  | |  | Advocate against other caregivers | | | | | | |
|  |  | |  | Other *(please specify)*: | | | | | | |
| 4. | How does hospice and palliative care staff (in your opinion) understand the role of volunteers related to patients and their family members? | | | | | | | | | |
|  |  | | | | | | | | | |
|  | a) *(Please choose one option only)* | | | | | | | | | |
|  |  | |  | Confidential friend | |  | | Neither nor |  | Stranger |
|  | b) *(Please choose one option only)* | | | | | | | | | |
|  |  | |  | Expert | |  | | Neither nor |  | Layperson |
|  | c) *(Please choose one option only)* | | | | | | | | | |
|  |  | |  | Part of the care team | |  | | Neither nor |  | Part of the family system |
|  | d) *(Multiple answers possible)* | | | | | | | | | |
|  |  | |  | Coordinator of hospice or palliative care | | | | | | |
|  |  | |  | Gatekeeper for hospice or palliative care | | | | | | |
|  |  | |  | Advocate against doctors | | | | | | |
|  |  | |  | Advocate against other caregivers | | | | | | |
|  |  | |  | Other *(please specify)*: | | | | | | |
| 5. | Please estimate how many volunteers offer professional skills for free (e.g. health care workers): | | | | | | | | | |
|  |  | | | | | | | | | |
|  |  | | Hardly any | | | | | | | |
|  |  | Some | | | | | | | | |
|  |  | About half | | | | | | | | |
|  |  | Most | | | | | | | | |
|  |  | Almost all | | | | | | | | |
|  |  | Other *(please specify)*: | | | | | | | | |
| 6. | Do volunteers receive compensations or other benefits in your country? | | | | | | | | | |
|  | *(Multiple answers possible)* | | | | | | | | | |
|  |  | | No | | | | | | | |
|  |  | Money | | | If yes: How much do volunteers receive? | | | | | |
|  |  |  | | | Amount per month: | | | | |  |
|  |  |  | | | Currency: | | | | |  |
|  |  |  | | | How often do they get paid? | | | | |  |
|  |  |  | | | Who makes the payment? | | | | |  |
|  |  | Provision of transport | | | If yes: Which type of transport? | | | | | |
|  |  |  | | |  | | Car | | | |
|  |  |  | | |  | | Bike | | | |
|  |  |  | | |  | | Other *(please specify)*: | | | |
|  |  | Meals | | |  | |  | | | |
|  |  | Goods | | |  | |  | | | |
|  |  | Medical care | | |  | |  | | | |
|  |  | Other compensations or benefits received by volunteers: | | | | | | | | |

| **III** | **Management, training and supervision** | | | | |
| --- | --- | --- | --- | --- | --- |
| 1. | Do any legal regulations exist concerning the volunteering/ community service in your country? *(Multiple answers possible)* | | | | |
|  |  | | | | |
|  |  | No | | | |
|  |  | Yes, there are legal regulations concerning volunteering/ community service in general | | | |
|  |  | Yes, there are legal regulations especially concerning volunteering/ community service for hospice or palliative care | | | |
| 2. | Do you have national standards for volunteers in your country?  *(Multiple answers possible)* | | | | |
|  |  | | | | |
|  |  | No | | | |
|  |  | Yes, there are national standards concerning volunteering/ community service in general | | | |
|  |  | Yes, there are national standards especially concerning volunteering/ community service for hospice or palliative care | | | |
| 3. | How are volunteers being managed/ coordinated? *(Multiple answers possible)* | | | | |
|  |  | | | | |
|  |  | No regular coordination | | | |
|  |  | Peer coordination by another volunteer | | | |
|  |  | Coordination by professional staff member | | | |
|  |  | Other *(please specify)*: | | | |
| 4. | How are volunteers being supervised? *(Multiple answers possible)* | | | | |
|  |  | | | | |
|  |  | No regular supervision | | | |
|  |  | Peer supervision by another volunteer | | | |
|  |  | Supervision by professional staff member | | | |
|  |  | Other *(please specify)*: | | | |
| 5. | Do volunteers receive training in your country? *(Multiple answers possible)* | | | | |
|  |  | | | | |
|  |  | No | | | |
|  |  | No, but start of training programme is planned | | | |
|  |  | Yes, training for volunteer work in hospice and palliative care | | | |
|  |  | Yes, training for bereavement work | | | |
|  |  | Other *(please specify)*: | | | |
| 6. | If volunteers receive training, when do they receive that training? | | | | |
|  | *(Multiple answers possible)* | | | | |
|  |  | Before the first assignment | | | |
|  |  | With the first assignment | | | |
|  |  | On a regular basis | | | |
| 7. | How many hours does the training cover? | | | |  |
|  |  | | | | |
| 8. | Which period of time (in weeks) does the training course take in total? | | | |  |
|  |  | | | | |
| 9. | Which topics are treated in the training course? | | | | |
|  |  | | | | |
| 10. | Who are the trainers? *(Multiple answers possible)* | | | | |
|  |  | | | | |
|  |  | Nurse |  | Experienced volunteer | |
|  |  | Physician/doctor |  | Other *(please specify)*: | |
|  |  | Social worker |  |  | |
| **IV** | **Change and challenges** | | | | |
| 1. | In your opinion, what is the benefit of having volunteers involved in hospice and palliative care? | | | | |
|  |  | | | | |
| 2. | In your opinion, which is the biggest challenge in the involvement of volunteers in hospice or palliative care? | | | | |
|  |  | | | | |
| 3. | Are there challenges with? *(Multiple answers possible)* | | | | |
|  |  | | | | |
|  |  | Cooperation with other volunteers | | | |
|  |  | Cooperation with professional staff | | | |
|  |  | Language barriers | | | |
|  |  | Unreliability | | | |
|  |  | Other *(please specify)*: | | | |
|  |  | | | | |
| 4. | How easy is it to find volunteers suitable to work in hospice and palliative care in your country? | | | | |
|  |  | | | | |
| 5. | Are there limitations to voluntary work in hospice or palliative care? Are there any tasks that volunteers cannot or are legally not allowed to perform? | | | | |
|  |  | | | | |
| 6. | In your opinion, what are the biggest changes (negative or positive) in volunteering in the past years that have affected hospice or palliative care? | | | | |
|  |  | | | | |
| 7. | What can the EAPC Task Force on Volunteering (European Association for Palliative Care)/ the APCA Research Network (African Palliative Care Association)^[[1]](#footnote-1)^ do to help to improve volunteering in hospice and palliative care in your country? | | | | |
|  |  | | | | |

| **V** | **Personal Details**  *For the evaluation of the survey we also need some information about yourself. This data is not used to identify you and your anonymity will not be affected.* | | | | | |
| --- | --- | --- | --- | --- | --- | --- |
| D1. | Your original profession? | | | | | |
|  |  | | | | | |
|  |  | Nurse |  | Management | | |
|  |  | Physician/doctor |  | Other *(please specify)*: | | |
| D2. | Your gender? | | | | | |
|  |  | | | | | |
|  |  | Female |  | Male | | |
| D3. | What is your year of birth? | | | |  | |
| D4. | How many years of professional work experience do you have? | | | |  | |
| D5. | Your nationality? | | | | |  |
| D6. | What kind of institution do you work for? | | | | | |
|  |  | | | | | |
|  |  | Palliative Care Unit |  | Home Care Service | | |
|  |  | Inpatient Hospice |  | National Association | | |
|  |  | Ministry of Health |  | Other *(please specify)*: | | |

*Thank you for your time and your participation and please feel free to tell us anything else about your experiences with volunteers on the backside of this page if you feel it has not been covered by the questionnaire. Results from this survey will be disseminated via the EAPC blog and eHospice Africa. If you want to be personally informed, please contact* [*s4caloth@uni-bonn.de*](mailto:s4caloth@uni-bonn.de) *(for African participants) or* [*kat.pabst88@freenet.de*](mailto:kat.pabst88@freenet.de) *(for European participants) to be included into the mailing list.*

|  | Any other comments/ experiences with volunteers? |
| --- | --- |
|  |  |
|  | In a second phase of our study, we want to ask people, who are working on a local level, for their experience with volunteers. Could you name us potential contact persons from your country and their email addresses? |

| **VI** | **Lottery**  *As you participated in our survey, you now have the chance to enter our lottery*  *and - with some luck- win an iPad mini.*  *The winner will be drawn randomly and notified via email, when we close the survey.* | | | | |
| --- | --- | --- | --- | --- | --- |
| 1. | Please enter your email address here, if you would like to participate. Your contact data will be stored separately from the questionnaire's data. | | | |  |
| 2. | Do you want APCA to include your contact information into a directory for further cooperations? | | | | |
|  |  | | | | |
|  |  | Yes |  | No | |

1. This survey is done in parallel in African and European countries. For African countries APCA is the primary point of contact, for European countries the EAPC. [↑](#footnote-ref-1)
